# Serosurveillance among urban slum and non-slum populations immunized with COVID-19 vaccines in Bangladesh

**DOI:** 10.1017/S0950268823001942

**Published:** 2024-01-05

**Authors:** Protim Sarker, Md Ahsanul Haq, Evana Akhtar, Anjan Kumar Roy, Md Biplob Hosen, Tarique Mohammad Nurul Huda, Sharmin Akter, Razu Ahmed, Md Razib Chowdhury, Jannatul Ferdous, Maya Vandenent, Mohammad Zahirul Islam, Rashid U. Zaman, Shams-El Arifeen, Abdur Razzaque, Rubhana Raqib

**Affiliations:** 1 International Center for Diarrhoeal Disease Research, Bangladesh (icddr,b), Dhaka, Bangladesh; 2 Department of Public Health, College of Public Health and Health Informatics, Qassim University, Al Bukairiyah, Saudi Arabia; 3 UNICEF, Dhaka, Bangladesh; 4 Embassy of Sweden in Bangladesh, Dhaka, Bangladesh; 5 British High Commission, Dhaka, Bangladesh

**Keywords:** children, adults, anti-spike-antibodies, mixed and match vaccination, SARS-CoV-2

## Abstract

Using two rounds of serosurveillance, we aimed to observe the COVID-19 vaccination status and the dynamics of antibody responses to different vaccines among urban slum and non-slum populations of Bangladesh. Adults (>18 years) and children (10–17 years) were enrolled in March and October 2022. Data including COVID-19 vaccine types and dosage uptake were collected. SARS-CoV-2 spike (S)-specific antibodies were measured in blood. The proportion of vaccinated children was significantly lower among slum than non-slum populations. Two doses of vaccines showed an increase in the level of anti-S-antibodies up to 2 months, followed by reduced levels at 2–6 months and a resurgence at 6–12 months. Children showed significantly higher anti-S-antibodies after two doses of the Pfizer–BioNTech vaccine than adults; however, after 6 months, the level of antibodies declined in younger children (10 - < 12 years). In a mixed vaccine approach, mRNA vaccines contributed to the highest antibody response whether given as the first two doses or as the third dose. Our findings emphasized the need for increasing the coverage of COVID-19 vaccination among slum children and booster dosing among all children. The use of mRNA vaccines in the mixed vaccination approach was found to be useful in boosting the antibody response to SARS-CoV-2.

## Introduction

As of May 2023, nearly 7 million deaths have been reported globally due to coronavirus disease 2019 (COVID-19), caused by severe acute respiratory syndrome coronavirus 2 (SARS-CoV-2) [1]. Vaccination is an effective public health tool, applied to prevent and control the spread of an epidemic or a pandemic. Several COVID-19 vaccines have been developed, evaluated, and rolled out within a very short period of time in response to the COVID-19 pandemic. As of now, 64% of the global population has been fully vaccinated [2].

In Bangladesh, the approval for COVID-19 vaccination was first given in January 2021, with ChAdOx1 nCoV-19 (COVISHIELD; AstraZeneca) being the first vaccine to be administered. Within the next 6 months, approvals were also given to Sputnik V (Gamaleya Research Institute of Epidemiology and Microbiology), Sinopharm BIBP (BBIBP-CorV, Sinopharm), BNT162b2 (Comirnaty, Pfizer–BioNTech), Sinovac (CoronaVac, Sinovac Biotech), Janssen Ad26.COV2-S (Johnson & Johnson), and lastly mRNA-1273 (Spikevax, Moderna) [3]. Initially, individuals aged ≥55 years were prioritized by the Directorate General of Health Services (DGHS) to receive the COVID-19 vaccine. The age bar was subsequently lowered to ≥40 years in February 2021, ≥30 years in July 2021, and university students in September 2021. The approval was extended to 12- to 17-year-old children in November 2021. To increase the protection against COVID-19, everyone aged 5 years and older was included in the vaccination programme in August 2022. Initially, vaccines were provided via enlistment through e-registration in a government web portal, where the national identification or birth certificate number is needed to verify the identity of an individual. To boost the rate of vaccine administration, mass vaccination was later introduced, which did not require any e-registration, but vaccination certificates were provided.

Sinopharm BIBP and Sinovac vaccines are inactivated vaccines; Pfizer–BioNTech and Moderna vaccines are mRNA-based vaccines; and AstraZeneca-COVISHIELD, Sputnik V, and Janssen vaccines are viral vector-based vaccines. Both the mRNA- and vector-based vaccines utilize spike (S) protein as the target immunogen [4]. Following vaccination and also after natural infection, the immune system responds by producing high levels of neutralizing antibodies against the S protein and demonstrates predictive protection against SARS-CoV-2 infection [5]. The monitoring of dynamics of immune responses generated after SARS-CoV-2 infection and by vaccination is important in controlling the spread of SARS-CoV-2 infection and disease management [6].

In this study, we conducted two rounds of serosurveillance at 6-month interval in five city corporations in Bangladesh. We compared the COVID-19 vaccination status among urban slum and non-slum populations in terms of the number of people vaccinated, types of vaccines, and number of doses received. We also determined S protein-specific antibody levels induced by vaccination as well as natural infection with SARS-CoV-2.

## Methods

### Study design and setting

In 2022, we conducted two rounds of cross-sectional serosurveillance at 6-month interval (March and October). The study enrolled residents of both sexes, aged 10 years or above, from urban slums and adjacent non-slum areas in Dhaka, Chattogram, Khulna, Sylhet, and Rangpur city corporations. These cities cover five out of eight divisions and represent the central, south-eastern, south-western, north-eastern, and north-western regions of the country, respectively.

### Sample size calculation

In a recently conducted serosurveillance, we found that the weighted seroprevalence of SARS-CoV-2 was 67.3% [7]. With a precision of 0.02% and a 95% confidence interval, a sample size of 2,113 was estimated for each round. Since a cluster sampling method was applied, we added a design effect of 1.5 to account for the increased variability introduced by clustering. Finally, assuming a 10% non-response rate, a sample size of 3,522 was estimated for each round.

### Selection of slum and non-slum areas and sampling strategy

We implemented a multi-stage sampling strategy using cluster sampling based on the probability proportional to size (PPS) method to select slums. The cumulative population size of slums for each city corporation in 2021–2022 was calculated, and the total number of residents was divided by the number of slum clusters to determine the sampling interval [8], which was consistent with the methodology used in other large surveys [9, 10]. A computer-generated random number between zero and the sampling interval was used to select the first slum from the cumulative population list. The sampling interval number was added to that random number to identify the second cluster, and this process was repeated to identify the remaining clusters [11]. For each selected slum, a nearby non-slum area with a similar population size and demographic characteristics was identified. Middle-class areas were identified considering some additional factors including housing conditions, income levels, educational attainment, and occupation from available data sources, i.e., the Bangladesh Bureau of Statistics and other relevant government agencies. We selected 11 slums and 11 non-slum clusters in each city corporation and enrolled 32 participants from each cluster.

### Selection of households and enrolment of study participants

Each slum and non-slum area was divided into several enumeration areas (EAs) consisting of approximately 120 households [12]. In a randomly selected EA, the serosurveillance started from the main connecting road. After selecting a household, eligible household members (maximum three members including at least one child) were approached and enrolled after obtaining consent. The next three households were skipped, and the fourth household with eligible participants was selected to make sure that the households selected were systematically spread across the EA. If there were no eligible participants in the selected household, members of the next household were selected. This way, the enrolment of households and participants continued until the required number of participants (n = 32) from that cluster was enrolled. If there were not enough participants in an EA, the next closest EA was visited. The inclusion criteria for enrolling participants were (1) male or female individuals aged ≥10 years and (2) potential participants and household heads signed the informed consent/assent form. Exclusion criteria were those (1) not willing to give blood samples and (2) with any conditions that, in the opinion of the investigator, might jeopardize the safety of study participants or interfere with the evaluation of the study objectives.

### Collection of data

A tablet-based structured questionnaire was utilized to collect data from both household heads and individuals, including socio-demographic information, data on current and past 6 months’ morbidity, specifically COVID-like symptoms or the presence of confirmed COVID-19 cases through RT-PCR test reports, and comorbidities (e.g. diabetes, hypertension, asthma, and overweight/obesity).

Data on the COVID-19 vaccination status were also collected from each individual that included information on whether they have received any COVID-19 vaccine or not, the reason for not taking the vaccine, the type of the vaccine taken, the number of doses received, and whether the vaccine was received through e-registration or mass vaccination. The data on the administration of vaccine shots, vaccine types, and number of doses received were verified by checking the vaccination certificates.

### Anthropometry and specimen collection

The height and weight of the enrolled participants were measured twice using a free-standing stadiometer (Seca 217, Hamburg, Germany) and a digital weighing scale (Camry-EB9063, China), respectively, and the body mass index (BMI) was calculated. Venous blood (10 ml) was collected at a single instance and centrifuged, and plasma was separated, aliquoted, and stored in a freezer at −20 °C. The frozen plasma samples were transported weekly in dry ice to the icddr,b Laboratory in Dhaka for storage at −80 °C for later use.

### Detection of SARS-CoV-2 spike (S) protein-specific antibodies

The Elecsys® Anti-SARS-CoV-2 S Immunoassay Kit (Roche Diagnostics GmbH, Mannheim, Germany) was used to determine the concentration of SARS-CoV-2 S protein-specific antibodies in plasma. The automated immunoassay analyser uses a double-antigen sandwich assay format to quantitatively measure high-affinity antibodies, including IgG, against the S protein receptor-binding domain (RBD). The assay provides a sensitivity of 98.8% and a clinical specificity of 99.98%. In comparison with a vesicular stomatitis virus (VSV)-based pseudo-neutralization assay, the Elecsys Anti-SARS-CoV-2 S assay has a positive agreement rate of 92.3%.

Antibody titres of a group of individuals were reported for different time intervals, that is, <2 months, 2–6 months, and >6–12 months after receiving the latest dose of any vaccine.

### Data analysis

Frequency tables were used to present the vaccination status and reasons for not receiving vaccination. A weighted population-based sampling method was utilized, which involved calculating the sum of two probabilities: the probability between clusters (p1) and the probability within clusters (p2). The sampling weight was determined by taking the inverse of the total probability for each chosen area (1/(p1 + p2)). To determine the odds ratio (OR) of vaccination in relation to key socio-demographic characteristics, a multiple logistic regression model was employed. S protein-specific antibody titres were skewed; thus, log transformation was performed to normalize the data. The multivariate regression model was used to estimate the geometric mean (GM) of S-IgG titres, and to compare between different time intervals after dosing, between adults and older (12–17 years) and younger (10 - < 12 years) children and between the various combinations of mixed and matched vaccination groups. The model was adjusted for different covariates, including age, sex, income, education, body mass index, locality (slum and non-slum), time difference between the last vaccine shot and blood collection, and sampling weight; division (Chattogram, Dhaka, Khulna, Rangpur, and Sylhet) was used as random factor. Statistical analysis was performed using STATA-15, and graphical charts were generated using GraphPad Prism 8.3.2.

## Results

### Socio-demographic characteristics

A total of 7,043 participants (3,521 in round I and 3,522 in round II of the serosurveillance) were enrolled from both slums and surrounding non-slum areas in five city corporations. The socio-demographic characteristics of the study population are given in Table 1. Among the slum and non-slum participants, the distribution of adults and children was similar in both rounds. The male-to-female ratio among adult participants was almost equal, and in children, the proportion of females was higher. The proportion of service holders, businessmen, homemakers, and students was higher among non-slum residents, while the proportion of transport drivers/owners/contractors and manual labourers was higher in slum areas. Many children, particularly in the slums, were found to stay at home and not going to school (n = 46 (6.7%) in round I and n = 54 (7.8%) in round II), although schools resumed regular activities during the surveillance period. The proportion of participants with longer years of formal education (≥6 years) and monthly income of BDT 40,000 or above (equivalent to USD 463 or more) was higher in non-slums than in slums.

**Table 1.** Socio-demographic characteristics of the study participants

	Round I (March 2022; *n* = 3,521)		Round II (October 2022; *n* = 3,522)	
	Slum (*n* = 1,756)	Non-slum (*n* = 1,765)	Slum (*n* = 1,762)	Non-slum (*n* = 1,760)
Age				
Overall	28.5 ± 15.6	29.7 ± 17.0	28.9 ± 16.1	30.6 ± 17.4
Adult (*n* = 4,278)	38.2 ± 12.8	40.4 ± 13.9	38.9 ± 12.8	41.2 ± 13.9
Children (*n* = 2,765)	13.7 ± 2.1	13.8 ± 2.2	13.2 ± 2.2	13.5 ± 2.2
Sex				
Overall				
Male	826 (47.0%)	853 (48.3%)	822 (46.7%)	819 (46.5%)
Female	930 (53.0%)	912 (51.7%)	940 (53.4%)	941 (53.5%)
*Adult*				
Male	529 (49.7%)	530 (50.3%)	538 (50.0%)	528 (48.7%)
Female	535 (50.3%)	523 (49.7%)	538 (50.0%)	557 (51.3%)
*Children*				
Male	297 (42.9%)	323 (45.4%)	284 (41.4%)	291 (43.1%)
Female	395 (57.0%)	389 (54.6%)	402 (58.6%)	384 (56.9%)
Occupation				
* Adult*				
Service	113 (10.6%)	208 (19.8%)	96 (8.9%)	142 (13.1%)
Business	107 (10.1%)	205 (19.5%)	160 (14.9%)	262 (24.2%)
Homemaker	338 (31.8%)	412 (39.1%)	364 (33.8%)	454 (41.8%)
Transport driver/owner/contractor	63 (5.9%)	13 (1.2)	56 (5.2%)	24 (2.2%)
Student	43 (4.0%)	121 (11.5%)	36 (3.3%)	93 (8.6%)
Manual labour job	311 (29.2%)	0	253 (23.5%)	0
Unemployed	65 (6.1%)	64 (6.1%)	80 (7.4%)	81 (7.5%)
Others	24 (2.3%)	30 (2.8%)	31 (2.9%)	29 (2.7%)
*Children*				
Student	549 (79.3%)	667 (93.7%)	558 (81.3%)	626 (92.7%)
No schooling/stay at home^a^	46 (6.6%)	11 (1.5%)	54 (7.87%)	25 (3.70%)
Service	29 (4.2%)	22 ((3.1%)	20 (2.9%)	13 (1.9%)
Homemaker	18 (2.6%)	10 (1.4%)	20 (2.9%)	10 (1.5%)
Manual labour job	47 (6.8%)	1 (0.14%)	27 (3.9%)	1 (0.15%)
Others	3 (0.43%)	1 (0.14%)	7 (1.0%)	0
Education				
*Adult*				
No formal education, years	330 (31.0%)	19 (1.8%)	357 (33.2%)	67 (6.2%)
1–5	344 (32.3%)	142 (13.5%)	347 (32.2%)	212 (19.5%)
6–10	298 (28.0%)	359 (34.1%)	302 (28.1%)	440 (40.6%)
11–12	65 (6.1%)	231 (21.9%)	41 (3.8%)	149 (13.7%)
>12	27 (2.5%)	302 (28.7%)	29 (2.7%)	217 (20.0%)
*Children*				
No formal education, years	30 (4.3%)	0	23 (3.35%)	1 (0.15%)
1–5	326 (47.1%)	173 (24.3%)	352 (51.3%)	212 (31.4%)
6–10	323 (46.7%)	485 (68.1%)	304 (44.3%)	428 (63.4%)
11–12	13 (1.9%)	52 (7.3%)	7 (1.0%)	31 (4.6%)
>12	0	2 (0.28%)	0	3 (0.44%)
BMI category				
*Adult*				
Underweight	140 (13.1%)	63 (6.0%)	163 (15.2%)	78 (7.2%)
Normal	576 (54.1%)	433 (41.1%)	554 (51.5%)	461 (42.5%)
Overweight	275 (25.9%)	390 (37.0%)	272 (25.3%)	393 (36.2%)
Obese	73 (6.9%)	167 (15.9%)	87 (8.1%)	153 (14.1%)
*Children*				
Underweight	415 (60.0%)	309 (43.4%)	428 (62.4%)	305 (45.2%)
Normal	234 (33.8%)	315 (44.2%)	229 (33.4%)	279 (41.3%)
Overweight	34 (4.9%)	67 (9.4%)	21 (3.1%)	70 (10.4%)
Obese	9 (1.3%)	21 (2.9%)	8 (1.1%)	21 (3.1%)
Household income (BDT)				
<20,000	1,139 (64.9%)	234 (13.3%)	1,057 (60.0%)	334 (19.0%)
20,000–40,000	541 (30.8%)	624 (35.4%)	654 (37.1%)	862 (49.0%)
41,000–70,000	76 (4.3%)	641 (36.3%)	51 (2.9%)	397 (22.6%)
>70,000	0	266 (15.1%)	0	167 (9.5%)
Smoking status				
Overall	586 (33.4%)	299 (16.9%)	496 (28.2%)	317 (18.0%)
Children	22 (3.2%)	6 (0.84%)	12 (1.7%)	7 (1.0%)
Adult	564 (53.0%)	293 (27.8%)	484 (45.0%)	310 (28.6%)
Vaccination method				
e-Registration	1,442 (99.5%)	1,520 (99.6%)	1,148 (72.7%)	1,409 (86.6%)
Mass vaccination	8 (0.55%)	6 (0.39%)	432 (27.3%)	217 (13.4%)

*Note*: Data are presented as the number (percentage) of participants or mean ± standard deviation (for age only).

Abbreviations: BDT, Bangladeshi taka; BMI, body mass index.

a No schooling/stay at home refers to children who neither go for formal schooling nor work for income generation.

### Vaccination status of study participants

In round I of the serosurveillance, more than 99% of vaccine recipients received COVID-19 vaccines through e-registration. In round II, 27.3% of slum and 13.4% of non-slum participants received vaccines through mass vaccination campaigns, and the rest received through e-registration. The rate of the vaccination, considering the receipt of at least one dose of the vaccine, remained similar among adults across the two rounds (94% and 96% in rounds I and II, respectively). However, among children (≤17 years), the vaccination rate increased in both slum (from 65% to 79%) and non-slum (from 73% to 87%) areas in 6 months (Table 2). A comparison between slum and non-slum populations showed that the vaccination rate was similar for adults, but for children, the rate was lower in slums in both rounds.

**Table 2.** Distribution of adult and child participants who received different doses of vaccines or remained unvaccinated

	Round I (March 2022)			Round II (October 2022)		
Vaccine doses	Slum	Non-slum	Total	Slum	Non-slum	Total
Adults	*n* = 1,064	*n* = 1,053	*n* = 2,117	*n* = 1,076	*n* = 1,085	*n* = 2,161
Single dose	220(20.7%)*	117(11.1%)	337(15.9%)^a^	115(10.7%)	51(4.70%)	166(7.68%)
Two doses	685(64.4%)	665(63.2%)	1,350(63.4%)	501(46.6%)	465(42.9%)	966(44.7%)
Three doses	92(8.65%)	224(21.3%)**	316(14.9%)	418(38.9%)	524(48.3%)*	942(43.6%)
Unvaccinated	67(6.30%)	47(4.46%)	114(5.38%)	42(3.90%)	45(4.15%)	87(4.03%)
Children	*n* = 692	*n* = 712	*n* = 1,404	*n* = 686	*n* = 675	*n* = 1,361
Single dose	119(17.2%)	128(18.0%)	247(17.6%)	206(30.0%)	185(27.4%)	391(28.7%)
Two doses	331(48.8%)	392(55.1%)*	723(51.5%)	334(48.7%)	397(58.8%)**	731(53.7%)
Three doses	3(0.43%)	0	3(0.21%)	6(0.87%)	4(0.59%)	10(0.73%)
Unvaccinated	239(34.5%)*	192(27.0%)	431(30.7%)^a^	140(20.4%)	89(13.2%)	229(16.8%)

*Note*: Data are presented as the number (percentage) of participants. The chi-square test was used to determine the significant differences between slum and non-slum participants; differences are considered significant when *P* < 0.05.

a Difference between round I and round II (*P* < 0.05).

* *P* < 0.05;

** *P* < 0.01.

In round I, the majority of the adult participants (79%) received two doses of COVID-19 vaccines, and 14% even received the third shot. During round II, third-dose recipients increased to 43%. The number of third-dose recipients was significantly higher in non-slum than in slum areas in both rounds (Table 2).

Analysis using the multiple logistic regression model across two rounds revealed that female individuals were 1.2 times more likely than male individuals to receive a COVID-19 vaccine in the overall population; in slums, the odds was 1.4, but no difference was noted among non-slum participants (Table 3). The probability of receiving vaccines was significantly higher among adults than among children in the overall population and in both slum and non-slum areas. Participants with longer years of formal education (>6 years) had about three times higher odds of receiving vaccines than participants with no formal education in both areas. Individuals with a higher income (>40,000 BDT) exhibited higher odds of receiving COVID-19 vaccines in the overall population, and in particular in the non-slum population (Table 3).

**Table 3.** Socio-demographic determinants of COVID-19 vaccine uptake among study participants

	Slum (*n* = 3,518)		Non-slum (*n* = 3,525)		Overall (*n* = 7,043)	
	OR (95% CI)	*P*-value	OR (95% CI)	*P*-value	OR (95% CI)	*P*-value
Sex						
Male	Ref.		Ref.		Ref.	
Female	1.38 (1.12, 1.70)	0.003	1.02 (0.80, 1.30)	0.871	1.20 (1.02, 1.39)	0.026
Age						
Children	Ref.		Ref.		Ref.	
Adult	8.50 (6.62, 11.1)	<0.001	5.93 (4.93, 8.00)	<0.001	7.32 (6.05, 8.94)	<0.001
Education						
No formal education	Ref.				Ref.	
1–5 years	0.80 (0.58, 1.13)	0.201	0.52 (0.20, 1.24)	0.176	0.73 (0.54, 0.99)	0.043
6–10 years	3.63 (2.46, 5.31)	<0.001	2.97 (1.14, 7.77)	0.026	3.82 (2.77, 5.31)	<0.001
>11 years	3.19 (1.22, 8.41)	0.018	3.74 (1.30, 10.7)	0.015	4.48 (2.64, 7.61)	<0.001
Household income (BDT)						
<20,000	Ref		Ref.		Ref.	
20,000–40,000	0.98 (0.79, 1.23)	0.883	1.22 (0.86, 1.73)	0.264	1.09 (0.91, 1.31)	0.307
40,000–70,000	0.73 (0.41, 1.30)	0.290	0.68 (0.48, 0.97)	0.035	0.71 (0.56, 0.90)	0.005
>70,000	–		0.44 (0.29, 0.67)	<0.001	0.46 (0.33, 0.64)	<0.001

*Note*: Multiple logistic regression model was used to estimate the *P*-value by including body mass index (category), age, sex, and household income in the same model. Sampling weight was considered as additional covariate.

Abbreviations: BDT, Bangladeshi taka; CI, confidence interval; OR, odds ratio.

A comparison of the vaccination status between different districts across the two rounds showed that the vaccination rate was much lower in Dhaka in both areas mainly due to the poor vaccination rate among children, which affected the overall rate (Supplementary Table S1). The rate of vaccination was the highest in Sylhet, followed by Chattogram. Notably, in Sylhet, 100% of adult and child participants received at least one dose of the vaccine in round II.

A small number of adults (114 (5.4%) in round I and 87 (4%) in round II) did not receive the vaccine shots. The proportion of unvaccinated children decreased substantially within the 6-month time interval in both slum and non-slum areas (Table 2). The reasons for not receiving vaccines were inability to register (unavailability of a national identity card or birth certificate, not receiving the message due to technical difficulty), vaccine hesitancy, age < 12 years, pregnancy, and chronic disease or disability (Supplementary Table S2). The reasons for vaccine hesitancy included (i) no belief in the existence of COVID-19; (ii) no faith in the COVID-19 vaccine efficiency, since many people got infected even after receiving the vaccine; (iii) religious belief that God will protect against all diseases; (iv) common belief among slum-dwellers that this was a disease of rich people.

### Spike antigen-specific antibody responses in relation to different doses, time, and COVID-19 vaccine types

When blood samples were collected from participants in a cross-sectional design, different participants were at different stages of vaccination: some had received a single dose, some received two or three doses, and some had not yet received the COVID-19 vaccine. Moreover, samples from different individuals were collected at different time intervals after dosing.

The geometric mean of S-IgG antibody titres after two or three doses was higher than that after a single dose. The scenario was the same in both adults and children (Table 4). Adults receiving different doses of vaccines as well as unvaccinated adults showed significantly higher antibody titres in round II than in round I. Antibody titres in unvaccinated children were significantly higher in round II than in round I; however, vaccine recipients showed a similar response in both rounds after a single dose and significantly decreased titres after two doses (Table 4).

**Table 4.** SARS-CoV-2 spike protein-specific antibody titres in adult and child participants receiving different doses of vaccines or remained unvaccinated

Vaccine dose	Round I (March 2022)	Round II (October 2022)	Percent change	*P*-value
Adults	*n* = 2,117	*n* = 2,161		
Single dose	1,599.6 ± 12.1	6,081.4 ± 13.9	380.0%	<0.001
Two doses	3,689.8 ± 19.8	7,762.5 ± 32.2	210.4%	<0.001
Three doses	4,909.1 ± 38.3	11,939.9 ± 31.5	243.2%	<0.001
Unvaccinated	1,600 ± 12.1	4,207.3 ± 10.4	263.0%	<0.001
Children^a^	*n* = 1,404	*n* = 1,361		
Single dose	8,279.4 ± 17.4	8,491.8 ± 21.0	102.6%	0.221
Two doses	13,551.9 ± 28.8	9,311.1 ± 21.0	−68.7%	0.004
Unvaccinated	979.5 ± 22.8	2,322.7 ± 16.4	237.1%	<0.001

*Note*: Data are presented as estimated mean ± standard deviation. Single blood samples were collected from participants to measure s-IgG titres. The multivariate regression model was used to estimate the geometric mean of S-IgG titres and to compare round I and round II; the model was adjusted by sex, income, education, body mass index, locality (slum and non-slum), time difference between the last vaccine shot and blood collection, and sampling weight; division (Chattogram, Dhaka, Khulna, Rangpur, and Sylhet) was used as random factor.

a Very few children received the third dose; thus, data are not shown.

**Figure 1.**
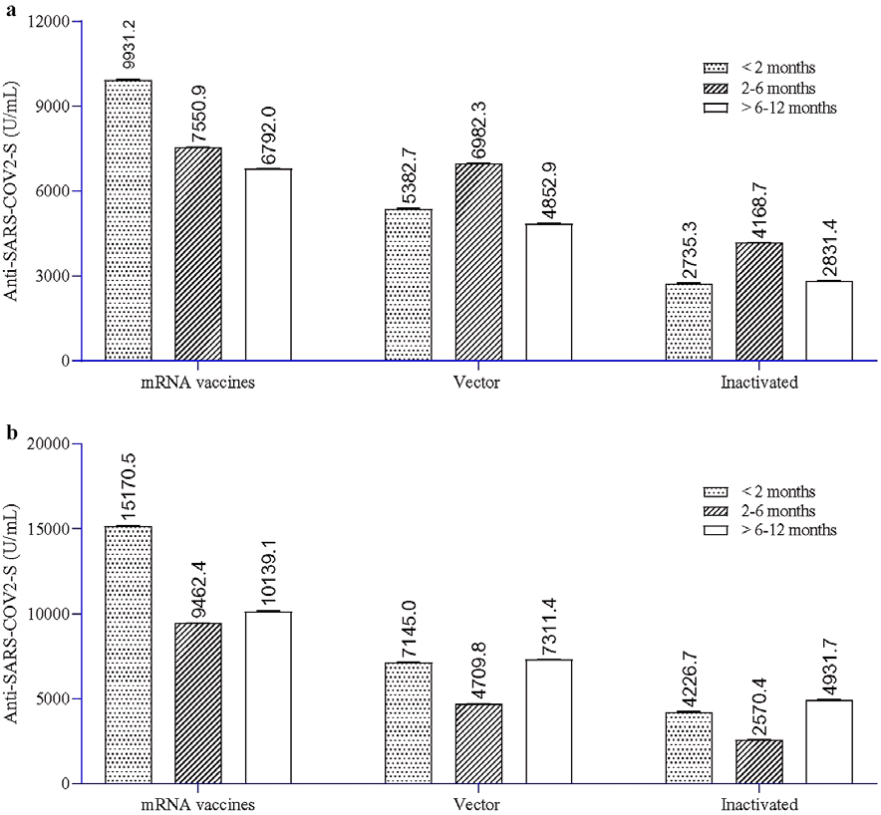
SARS-CoV-2 spike (S) protein-specific IgG titres (geometric mean) at different time intervals (<2 months, 2–6 months, and > 6–12 months) from the time of receipt of the last dose of different types of COVID-19 vaccines: (a) S-IgG titres in single-dose recipients and (b) S-IgG titres in two-dose recipients. Single blood samples were collected from participants to measure S-IgG titres. The multivariate regression model was used to estimate the geometric mean (GM) and to compare different time intervals after dosing; the model was adjusted for age, sex, income, education, body mass index, locality (slum and non-slum), and sampling weight; division (Chattogram, Dhaka, Khulna, Rangpur, and Sylhet) was used as random factor.

Comparisons were also made in antibody titres between different vaccines at different time intervals (<2 months; 2–6 months; >6–12 months) after receiving any dose of vaccine. At any given dose (single or double) and at any time interval, mRNA vaccines mounted a higher antibody response than vector-based or inactivated vaccines (Figure 1). Among mRNA vaccine recipients after a single dose of vaccination, an initial increase of S-IgG GM titres up to <2 months was followed by a gradual reduction at 2–6 months and > 6–12 months. Both vector-based and inactivated vaccine recipients showed elevated GM titres of S-IgG at a 2- to 6-month interval compared to <2 months; however, antibody titres decreased at >6–12 months. Following two doses of vaccines, antibody titres among any type of vaccine recipients were the highest up to 2 months, then declined at the 2- to 6-month interval, and again elevated at >6–12 months (Figure 1).

### Comparison of S-IgG titres between adults and children

**Figure 2.**
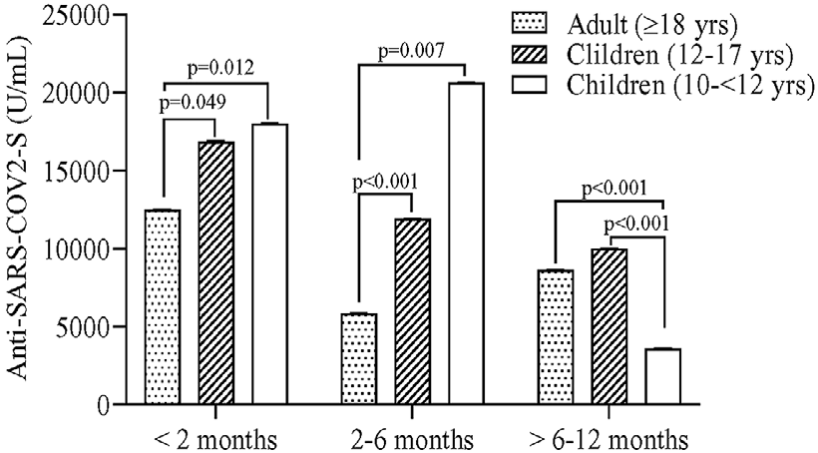
SARS-CoV-2 spike (S) protein-specific IgG titres in adults and children at different time intervals (<2 months, 2–6 months, and > 6–12 months) after administration of two doses of the Pfizer–BioNTech vaccine. Single blood samples were collected from participants to measure S-IgG titres. The multivariate regression model was used to estimate the geometric mean of S-IgG titres and to compare adults and older (12–17 years) and younger (10 - < 12 years) children; the model was adjusted for sex, income, education, body mass index, locality (slum and non-slum), time difference between the last vaccine shot and blood collection, and sampling weight; division (Chattogram, Dhaka, Khulna, Rangpur, and Sylhet) was used as random factor.

S-IgG antibody titres were compared between adult and child recipients of the Pfizer–BioNTech vaccine. Both the younger (10 - < 12 years) and older (12–17 years) children showed significantly higher S-IgG GM titres than adults at <2 months and 2–6 months following the second dose of vaccination. After 6 months, S-IgG titres were similar in adults and older children but were found to be significantly lower in younger children (Figure 2). No such pattern was observed after a single dose of vaccine.

### S-IgG response in matched and mixed vaccination

**Figure 3.**
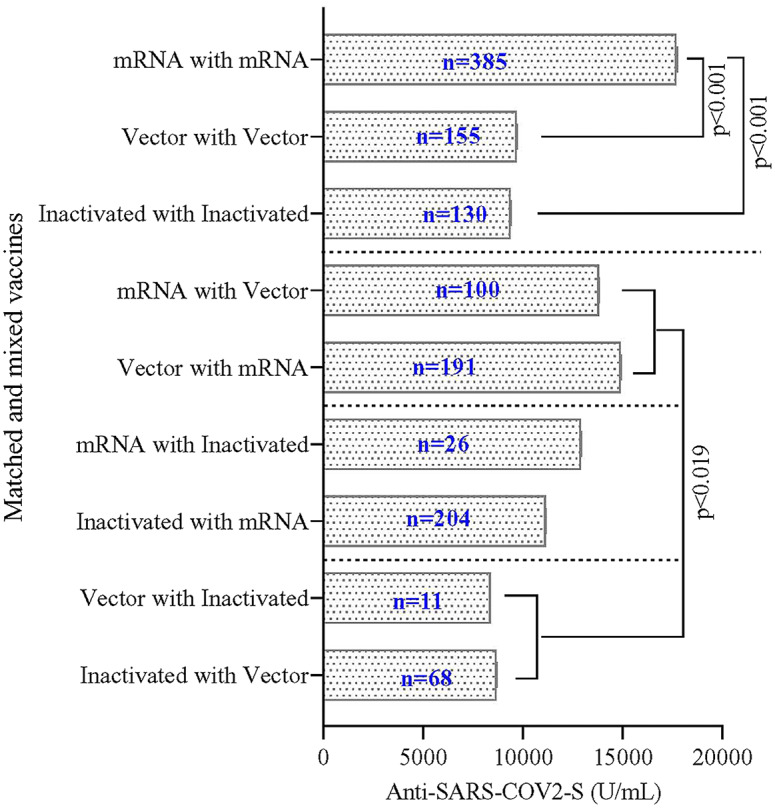
SARS-CoV-2 spike (S) protein-specific IgG titres in matched and mixed vaccine recipients. ‘Matched vaccination’ refers to immunization with the same vaccine at the first, second, and third dosing, and ‘mixed vaccination’ refers to the same vaccine given at the first and second dosing, while a different vaccine is given at the third dosing. Single blood samples were collected from participants to measure S-IgG titres. The multivariate regression model was used to estimate the geometric mean of S-IgG titres and to compare the various combinations of mixed and matched vaccination groups. The model was adjusted for age, sex, income, education, body mass index, locality (slum and non-slum participants), time difference between the last vaccine shot and blood collection, and sampling weight; division (Chattogram, Dhaka, Khulna, Rangpur, and Sylhet) was used as random factor.

A comparison between matched vaccination, that is, immunization with the same vaccine at the first, second, and third dosing, demonstrated a significantly higher antibody concentration in mRNA vaccine recipients than in vector-based and inactivated vaccine recipients (Figure 3).

Mixed vaccination refers to the same vaccine given at the first and the second dosing, while a different vaccine is given at the third dosing. We found that the mRNA vaccine contributed the highest level of antibody titres whether given as the first two doses boosted with a different vaccine or given as the third dose after primary dosing with either a vector-based or an inactivated vaccine (Figure 3).

## Discussion

In the serosurveillance study, there was little increase in the rate of COVID-19 vaccination among adults over 6 months as the vaccination rate was already high (94% in round I and 96.0 in round II), while the rate increased substantially in children as more and more children were enrolled into the immunization programme through their schools or local municipality. In both rounds, the distribution of vaccinated adults was similar in slum and non-slum areas, while the proportion of vaccinated children was significantly lower in slum areas. Post-vaccination induction and the persistence of S-IgG varied depending on the vaccine types, number of doses of vaccines received, and the time interval of antibody measurement following the vaccination. Children showed significantly higher S-IgG titres than adults after receiving two shots of the Pfizer–BioNTech vaccine. Regarding booster dosing, mRNA vaccines given as three doses (matched vaccination) or combined with vector or inactivated vaccines (mixed vaccination) generated a higher antibody response than vector or inactivated vaccines when used in matched and mixed vaccination strategies.

The serosurveillance study demonstrated an increase in the COVID-19 vaccination coverage in the overall study population from 85% to 91% in 6 months, and the increase was mainly due to the increase in the vaccination rate among children. It was also found that in round I, almost all the vaccine recipients received vaccines through e-registration, while in round II, 20.4% of the vaccination was provided through mass vaccination. The campaign to vaccinate 12- to 17-year-old children started in schools in November 2021. Initially, only the parents of school-going children were aware of the campaign/programme. Gradually when announcements were made through megaphones in the streets, the rate of vaccination among children increased substantially. Vaccination in children further increased, when 10- to 12-year-old children started getting registered after the government announcement regarding inclusion of younger children (5–11 years) in August 2022. The vaccination rate among children (69%) was much lower than that among adults (94%) in round I, which is quite expected as vaccination in children started much later than that in adults. Despite the increase in the vaccination rate at the 6-month interval, the proportion of children receiving the vaccine did not reach the level among adults and was lower in slum than in non-slum areas. One reason could be that a large number of slum children did not go to school during the surveillance period and missed the vaccination. These findings emphasize that more children, particularly in slums, need to be brought under vaccine coverage.

Despite strong recommendations for COVID-19 vaccination to reduce mortality and severity of disease and generate herd immunity against COVID-19, there are wide variations in vaccine acceptance between countries and between different populations. In many countries including the USA and some European countries, vaccine acceptance is quite low [13, 14]. When the COVID-19 vaccine was first introduced in February 2021, vaccine hesitancy was also a major issue in Bangladesh [15, 16]. The common factors related to unwillingness to receive vaccines were concerns about side effects, presumed poor vaccine quality, biological/genetic materials used in vaccine manufacturing, and scepticism of vaccine efficacy. Vaccine hesitancy was mostly related to younger age (18–25 years), male gender, low income, low education, and unemployment. Among the educated class, there were concerns that COVID-19 vaccines were developed very rapidly unlike the standard procedure that usually takes several years and were given emergency use approvals because of the precarious pandemic situation; there was not enough time to gather evidence about long-term adverse events of vaccination [15, 16]. Moreover, new information was emerging about the severity of COVID-19 disease in people with noncommunicable diseases (NCDs). There was still a lack of information about the suitability of these vaccines among pregnant women or elderly individuals and patients with NCDs and other underlying diseases [17]. However, the scenario changed, and gradually, the level of vaccine acceptance increased, which was also reflected in our study population. Very few unvaccinated participants remained, who were eligible to receive the vaccine. However, in Dhaka, the capital city of Bangladesh, the vaccination rate was much lower in both slum and non-slum areas than in other city corporations. A possible explanation could be that the massive population living in the sprawling mega-city Dhaka is mostly occupied with earning livelihood and has little time for or gives less importance to vaccination.

As expected, the antibody response was higher among vaccinated adults and children than among unvaccinated participants, and a dose response was seen in both age groups. An increase in antibody titres in unvaccinated adults and children in a span of 6 months may suggest increased rates of exposure and infection. The antibody response generated by the mRNA vaccine was much higher than that generated by vector-based or inactivated vaccines, which is in line with the previous reports from us and other groups [18–23]. When the time interval between the latest vaccine dose and antibody measurement was considered, a decreasing trend of the antibody titre was seen from two months onward after a single dose of mRNA vaccine. In contrast, antibody titres were elevated in both vector-based and inactivated vaccine recipients at 2–6 months. It is likely that among mRNA vaccine recipients, there were no breakthrough infections, while this was not the case with the other two vaccine types in the selected population. We further observed that after two doses of vaccination, following a decline at 2–6 months, there was a resurgence of antibodies at >6–12 months in all vaccine recipients. We may hypothesize that the decline in antibody levels below a threshold level did not protect against future infections, giving rise to increased levels of antibodies again; however, we did not have data to show whether the infection resulted in asymptomatic condition, mild disease, or moderate disease.

Data are limited on comparisons of COVID-19 vaccine responses in adults and children in the community; most comparative studies are focused on immune response to SARS-CoV-2 infection. A number of studies described that adults produce a broader set of antibodies, which include more virus-neutralizing antibodies, than children despite having similar levels of viral loads in nasopharyngeal/throat swabs and similar mild symptoms among the age groups [24, 25]. Growing evidence suggests that children exhibit a low adaptive immune response that targets immune memory (memory B and T cells), instead they mount a stronger and faster innate immune response to SAR-CoV-2 infection (e.g. naïve T cells) [25, 26]. It is likely that the initial robust innate immune response in children to clear the virus may hamper the development of effective adaptive cell-mediated immunity and their ability to resist future infections [27]. However, one study in Italy has reported that young children (≤3 years) showed significantly higher (fivefold) S-RBD IgG antibody titres than adults during mild symptomatic infections [28]. Here, we have demonstrated that repeated doses of the Pfizer vaccine generated a higher S-IgG antibody response in children than in adults. However, the faster decline in antibody titres in younger children than in older children and adults emphasizes the need for additional/booster dosing in younger children. Further studies are warranted to confirm the findings and to understand the longevity of B and T memory-cell responses after COVID-19 vaccination in young children in a longitudinal follow-up study.

The Government of Bangladesh initiated the booster dose in December 2021, and because of the uncertainty about the types of COVID-19 vaccines being available during the pandemic, mixing of vaccines was allowed during the third dose. A number of studies have reported higher antibody levels when mRNA vaccines were included in the combination [29–32]. Our findings were in the same direction; combinations of mRNA vaccines with vector-based or inactivated vaccines showed higher antibody responses than matched or mixed non-mRNA vaccines. These findings may have policy implications; cheaper vaccines can be used for two preliminary doses, followed by boosting with mRNA vaccines to generate a strong protective immune response.

Due to the short duration of protection provided and a rapid decline in efficacy, repeated doses of COVID-19 vaccination have been encouraged. However, there are recommendations for vulnerable, elderly individuals to be prioritized for additional boosters of COVID-19 vaccines [31, 32]. Many studies have reported exhaustion of immunity after third to fifth doses of the vaccines, particularly among patients with underlying health conditions and those receiving mRNA vaccines [22, 23]. However, repeated immunization with mRNA vaccines has been shown by another study group to induce high levels of IL-17, eliciting a strong inflammatory response and disrupting the Th1–Th2 immune balance. Moreover, mRNA vaccines failed to induce effector memory T cells in the vulnerable population [33]. Repeated stimulatory conditions promoted naïve T-cell differentiation towards a pro-inflammatory phenotype and suppressed IFN-γ production by SARS-CoV-2-specific CD4 + Th1 cells [34]. Therefore, personalized vaccination has also been proposed to be a useful strategy, whereby target populations are identified to receive booster doses, who would benefit the most. However, the applicability of such an approach in resource-poor countries is yet to be seen.

Accumulating evidence shows that COVID-19 vaccination has been associated with adverse events following immunization, especially on the nervous [35] and cardiovascular systems [36, 37] in different age groups and with sex and gender differences [38]. However, clear-cut associations have not been established [36, 39]. Now that the pandemic emergency has passed, in-depth longitudinal vaccine safety surveillance data from real-life settings are essential for determining the true safety profile, identifying the factors causing the adverse events, and improving vaccine formulations [40, 41].

The study has a number of limitations. We could not confirm the intermittent SARS-CoV-2 infections between vaccine doses by molecular testing for many participants, which would provide evidence of the effect of natural infection on the kinetics of antibody responses generated by vaccines. There were multiple reasons for the low rate of molecular testing for SARS-CoV-2 among the participants – poor public awareness about the importance of continued testing for COVID-19, the common perception that vaccination would provide sufficient protection against the disease, high testing costs, and some travel- and workplace-related hindrance. We did not measure N-specific antibody titres that could distinguish between N-specific antibodies induced by vaccination from those generated by natural infection with SARS-CoV-2. One main reason was that a significant proportion of study participants (n = 1864) received inactivated vaccines, and excluding this group from analysis could introduce a significant bias. Another reason was funding constraints. The study only covers urban areas in five divisions and thus is not nationally representative. The scope of the study was limited to congested city areas where people were most likely to spread the SARS-CoV-2 infection due to poor and unhygienic living conditions and at the same time likely to get less focus for vaccination. Middle-class families from neighbourhoods of slum areas were also included for comparing infection and vaccination status between the two populations. The inclusion of rural communities would have enabled a broader nation-wide picture, but was not possible due to limited resources. However, the strength of the study was sampling weights, for example, locality (equal proportions of slum and non-slum participants), sex (equal ratio 1:1), and age categories (adults and children, 60:40 ratio) that were used to generate representative results from five divisions of Bangladesh [8].

The findings of the study underscore the need for focused attention to enhance COVID-19 vaccine coverage in slum children. A significant reduction in S-antibody titres in the younger age group after 6 months of receiving vaccines reflects the need for booster doses in this age group. Combining mRNA vaccines with other COVID-19 vaccines in a mixed vaccination approach could be a strategy of choice to maximize the immune response and prolong protection against SAR-CoV-2 infection.

## Supporting information

Sarker et al. supplementary material Sarker et al. supplementary material

## Data Availability

The data that support the findings of this study are available upon request.

## Supplementary material

For supplementary material accompanying this paper visit http://doi.org/10.1017/S0950268823001942. click here to view supplementary material
