# Supplementary material for: Serosurveillance among urban slum and non-slum populations immunized with COVID-19 vaccines in Bangladesh
Source: Epidemiol Infect. 2024 Jan 5;152:e14. doi: 10.1017/S0950268823001942 (PMC10804132; doi:10.1017/S0950268823001942)
Supplement: Sarker et al. supplementary material [file S0950268823001942sup001.docx]

**Serosurveillance among urban slum and non-slum population immunized with COVD-19 vaccines in Bangladesh**

Protim Sarker^1^, Md. Ahsanul Haq^1^, Evana Akhtar^1^, Anjan Kumar Roy^1^, Md. Biplob Hosen^1^, Tarique Mohammad Nurul Huda^1†^, Sharmin Akter^1^, Razu Ahmed^1^, Md. Razib Chowdhury^2^, Jannatul Firdaus^3^, Maya Vandenent^3^, Zahirul Islam^4^, Rashid U. Zaman^5^, Shams-El Arifeen^1^, Abdur Razzaque^1^, Rubhana Raqib^1*^

^1^Infectious Diseases Division, International Center for Diarrhoeal Disease Research, Bangladesh (icddr,b), Dhaka-1212, Bangladesh; ^3^UNICEF, Dhaka-1207, Bangladesh; ^4^Embassy of Sweden in Bangladesh, Dhaka-1212, Bangladesh; ^5^British High Commission, Dhaka-1212, Bangladesh;

^*^**Corresponding author:**

Rubhana Raqib

Email: [rubhana@icddrb.org](mailto:rubhana@icddrb.org)

^†^Present Address: Department of Public Health, College of Public Health and Health Informatics, Qassim University, Al Bukairiyah 52741, Saudi Arabia

**Supplementary Table 1.** Distribution of participants receiving COVID-19 vaccines in different city corporations^1^.

|  | Chattogram (n=1393) | | Dhaka (n=1417) | | Khulna (n=1425) | | Rangpur (n=1397) | | Sylhet (n=1411) | |
| --- | --- | --- | --- | --- | --- | --- | --- | --- | --- | --- |
|  | Round I | Round II | Round I | Round II | Round I | Round II | Round I | Round II | Round I | Round II |
| Overall | (n=702)  581(82.8%) | (n=691)  639(92.5%) | (n=708)  546(77.1%) | (n=709)  556(78.4%) | (n=706)  634(89.8%) | (n=719)  657(91.4%) | (n=708)  609(86.0%) | (n=689)  640(92.9%) | (n=697)  606(86.9%) | (n=714)  714(100%) |
| Adults | (n=417)  384(92.1%) | (n=436)  417(95.0%) | (n=440)  406(92.3%) | (n=436)  389(89.2%) | (n=422)  413(97.9%) | (n=439)  427(97.3%) | (n=421)  406(96.4%) | (n=415)  406(97.8%) | (n=417)  394(94.5%) | (n=435)  435(100%) |
| Children | (n=285)  197(69.1%) | (n=255)  222(87.1%) | (n=268)  140(52.2%) | (n=273)  167(61.2%) | (n=284)  221(77.8%) | (n=280)  230(82.1%) | (n=287)  203(70.7%) | (n=274)  207(75.5%) | (n=280)  212(75.7%) | (n=279)  279(100%) |

Data are presented as number (percentage) of participants. ^1^Participants receiving at least a single dose of COVID-19 vaccines are included**.**

**Supplementary Table 2.** Reasons for not getting vaccinated

| Reasons | Round I (March 2022; n=545) | Round II (October 2022; n=316) | Total (n=861) |
| --- | --- | --- | --- |
| Inability to e-register^1^ | 174 (31.9%) | 88(27.8%) | 262 (30.4%) |
| Vaccine hesitancy | 158 (29.0%) | 132(41.8%) | 290 (33.7%) |
| Chronic disease, disability or physical problem | 13 (2.4%) | 18(5.7%) | 31 (3.6%) |
| Pregnancy | 12 (2.2%) | 15(4.7%) | 27 (3.1%) |
| Age <12 years^2^ | 188 (34.5%) | 63(19.9%) | 251 (29.2%) |

Data are presented as number (percentage) of participants. ^1^E-registration in a government web portal (‘Shurokkha’ Application) requires possession of national identity (NID) card or birth certificate; registered participants are invited to receive vaccines through Short Message Service (SMS). ^2^The announcement for inclusion of children aged 5-11 years in the COVID-19 vaccination program was made on 25 August 2022.
